# Longitudinal characterization of determinants associated with obesogenic growth patterns in early childhood

**DOI:** 10.1093/ije/dyac177

**Published:** 2022-09-10

**Authors:** Navin Michael, Varsha Gupta, Anna Fogel, Jonathan Huang, Li Chen, Suresh Anand Sadananthan, Yi Ying Ong, Izzuddin M Aris, Wei Wei Pang, Wen Lun Yuan, See Ling Loy, Mya Thway Tint, Kok Hian Tan, Jerry KY Chan, Shiao-Yng Chan, Lynette Pei-Chi Shek, Fabian Yap, Keith Godfrey, Yap Seng Chong, Peter Gluckman, S. Sendhil Velan, Ciarán G. Forde, Yung Seng Lee, Johan G. Eriksson, Neerja Karnani

**Affiliations:** 1 Singapore Institute for Clinical Sciences, Agency for Science Technology and Research, Singapore; 2 Department of Pediatrics, Yong Loo Lin School of Medicine, National University of Singapore, Singapore; 3 Division of Chronic Disease Research Across the Lifecourse, Department of Population Medicine, Harvard Medical School and Harvard Pilgrim Health Care Institute, Boston, USA; 4 Department of Obstetrics and Gynaecology and Human Potential Translational Research programme, Yong Loo Lin School of Medicine, National University of Singapore, Singapore; 5 Department of Reproductive Medicine, KK Women’s and Children’s Hospital, Singapore; 6 Duke-NUS Medical School, 8 College Road, Singapore; 7 Department of Maternal Fetal Medicine, KK Women's and Children's Hospital, Singapore; 8 Academic Medicine, Duke-National University of Singapore Graduate Medical School, Singapore; 9 Department of Reproductive Medicine, KK Women's and Children's Hospital; 10 Department of Pediatrics, KK Women’s and Children’s Hospital, Singapore; 11 Lee Kong Chian School of Medicine, Nanyang Technological University, Singapore; 12 Liggins Institute, University of Auckland, Auckland, New Zealand; 13 Institute of Bioengineering & Bioimaging, Agency for Science Technology and Research, Singapore; 14 Division of Human Nutrition and Health, Wageningen University & Research, Wageningen, Netherlands; 15 Department of General Practice and Primary Health Care, University of Helsinki, Finland; 16 Folkhälsan Research Center, Helsinki, Finland; 17 Department of Biochemistry, Yong Loo Lin School of Medicine, National University of Singapore, Singapore; 18 MRC Lifecourse Epidemiology Centre and NIHR Southampton Biomedical Research Centre, University of Southampton and University Hospital Southampton NHS Foundation Trust, Southampton, UK; 19 Bioinformatics Institute, Agency for Science Technology and Research, Singapore; 20 Université de Paris, CRESS, Inserm, INRAE, F-75004 Paris, France

**Keywords:** Childhood obesity, mother-offspring cohort, growth trajectories, BMI-z-score trajectories, group-based trajectory modeling, risk factors for childhood obesity

## Abstract

**Background:**

Longitudinal assessment of the determinants of obesogenic growth trajectories in childhood can suggest appropriate developmental windows for intervention.

**Methods:**

Latent class growth mixture modelling was used to identify body mass index (BMI) z-score trajectories from birth to age 6 years in 994 children from prospective mother-offspring cohort (Chinese, Indian and Malay ethnicities) based in Singapore. We evaluated the early life determinants of the trajectories as well as their associations with cardiometabolic risk markers at age 6 years.

**Results:**

Five BMI z-score trajectory patterns were identified; three within the healthy weight range, alongside early-acceleration and late-acceleration obesogenic trajectories. The early-acceleration pattern was characterized by elevated fetal abdominal circumference growth velocity, BMI acceleration immediately after birth, and crossing of the obesity threshold by age 2y. The late-acceleration pattern had normal fetal growth, BMI acceleration after infancy, and approached the obesity threshold by age 6 years. Abdominal fat, liver fat, insulin resistance, and odds of prehypertension/hypertension were elevated in both groups. Indian ethnicity, high pre-pregnancy BMI, high polygenic risk scores for obesity and shorter breastfeeding duration were common risk factors for both groups. Malay ethnicity and low maternal educational attainment were uniquely associated with early BMI acceleration, while nulliparity and obesogenic eating behaviors in early childhood were uniquely associated with late BMI acceleration.

**Conclusion:**

BMI acceleration starting immediately after birth or after infancy were both linked to early cardiometabolic alterations. The determinants of these trajectories may be useful for developing early risk stratification and intervention approaches to counteract metabolic adversities linked to childhood obesity.

## Introduction

Accelerated growth in infancy and early childhood have been linked to obesity and impaired cardiometabolic health in adulthood^1–4^. Nearly 90% of children who are overweight/obese in early childhood continue to be overweight/obese in adolescence^4^. Unlike the risk for diabetes, the risk for coronary disease linked to adolescent obesity is not attenuated even if normal weight is achieved by adulthood, highlighting the importance of tracking childhood growth patterns^5^. Given the rising rates of childhood obesity in Asia^6^, a better understanding of the heterogeneity of obesogenic growth patterns in Asian populations, and of the prenatal and postnatal factors that drive them, can bring precision to international guidelines and preventive strategies being developed to tackle childhood obesity^7^.

In the Growing Up in Singapore Towards healthy Outcomes (GUSTO) prospective mother-offspring cohort^8^, latent class growth mixture modelling (LCGMM) was used to identify group-based heterogeneous body mass index (BMI) trajectory patterns between birth and age 6 years. LCGMM allows group-based classification of developmental trajectories based on shared growth patterns, while allowing for within-class heterogeneity. We evaluated the associations of growth patterns with prenatal and postnatal risk factors, fetal growth, genetic risk of obesity, obesogenic eating behaviours, abdominal and ectopic fat (liver fat and intramyocellular lipids) depots, and cardiometabolic risk markers in early childhood.

## Methods

### Study Population

The GUSTO cohort recruited 1450 (≥18y of age) Chinese, Indian and Malay women with homogenous parental ethnicities, in the first trimester from two major public maternity hospitals in Singapore^8^. Exclusion criteria for the current analysis were preterm birth (< 37 weeks), multiple pregnancies or absence of at least one BMI assessment between birth and age 6 years. The final study sample consisted of 994 children. Owing to differential consent for imaging, cardiometabolic and behavioral assessments, these data were available in subsets of children as indicated in supplementary Fig. S1.

### Maternal and Intrauterine Measurements

We obtained information regarding ethnicity, parity, education, pre-pregnancy weight, and age from questionnaires administered upon study enrollment in the first trimester. Pre-pregnancy BMI (ppBMI) was computed from maternal height measured during the 26-28^th^ week of pregnancy and self-reported pre-pregnancy weight. At 26-28^th^ weeks, gestational diabetes mellitus (GDM) status was assessed using a 2-hr 75 g oral glucose tolerance test^9^ based on the World Health Organization (WHO) 1999 guideline^10^. Information about hypertensive disorders of pregnancy (pregnancy induced hypertension, preeclampsia or eclampsia) were obtained from medical records. Serial weight measurements during pregnancy were used to calculate the linear rate of gestational weight gain (GWG) between 15-35 weeks gestation^11^. This was used to classify women into insufficient, normal or excessive rate of gestation weight gain categories based on the Institute of Medicine (IOM) 2009 guidelines^12^. Fetal abdominal circumference (FAC) was measured from ultrasound scans in the 2nd (week 19-21) and 3rd (week 32-34) trimesters on standard views at the level of the stomach, where the umbilical vein enters the portal sinus. FAC velocity (FACV) was defined as the rate of change of FAC between 2nd and 3rd trimester in mm per week.

### Polygenic Risk Scores for Obesity

Umbilical cord DNA samples from the children were genotyped using the Illumina OmniExpress + exome array covering ~1 million SNPs. The genotype data was used to derive polygenic risk scores (PRS) for obesity as described earlier^13^.

### Breastfeeding and Eating Behaviors Assessments

Breastfeeding duration data^14^ was recorded as a dichotomous variable: < 3 vs ≥ 3 months of any (exclusive, predominant or partial) breastfeeding. Objectively measured child eating behaviors (eating rate (g/min), chewing (cycles/g), oral exposure time (s), energy intake (kcals) and eating in absence of hunger) were measured during an *ad libitum* laboratory lunchtime meal at age 4.5y as described previously^15^.

### Cardiometabolic Assessments

Fasting blood collected at age 6 years was used to measure plasma glucose using the enzymatic hexokinase method and serum insulin using a sandwich immunoassay. Fasting glucose and insulin were used to calculate the homeostatic model assessment of insulin resistance (HOMA-IR)^16^. At age 6 years, having either prehypertension or hypertension was diagnosed using the simplified pediatric blood pressure threshold of 110/70 mmHg which has been shown to perform as well as age, sex and height-standardized thresholds for predicting adulthood cardiovascular risk^17^.

### Imaging of Body Fat Depots

Subcutaneous adipose tissue (SAT) and intra-abdominal adipose tissue (IAT) volumes in children were assessed using magnetic resonance imaging (MRI) during the first 2 weeks after birth and at age 6 years^18, 19^. Magnetic resonance spectroscopy (MRS) was performed to measure intramyocellular lipids (IMCL) in soleus muscle and liver fat in the children at the age of 6 years (detailed MRS protocols are provided in the supplementary).

### Modeling of BMI z-score Trajectories

Serial BMI measurements (birth, 3 weeks, 3m, 6m, 9m, 12m, 15m, 18m, 24m, 36m, 48m, 54m, 60m, 66m & 72m) were converted to age and sex standardized z-scores based on the 2006 World Health Organization (WHO) Child Growth Standards using the WHO Anthro macro for SPSS (v3.2.2, Jan 2011)^20^. Latent class growth mixture modelling (LCGMM) was used to derive heterogeneous groups of BMI z-score trajectories using Mplus Version 8^21^ (detailed protocols for anthropometric assessments and LCGMM analysis are provided in the supplementary).

### Statistical analysis

Statistical analyses were performed using IBM SPSS Statistics v23. Multiple imputation by fully conditional specification was used to handle missing values in exposures and covariates. The imputation model included all exposures, covariates and postnatal cardio-metabolic assessments as predictors. Multinomial logistic regression was used to study the association of maternal antenatal exposures with the postnatal trajectory classification in a mutually adjusted model. Two additional multinomial logistic regression models were constructed to study the associations of breastfeeding duration (adjusted for ethnicity, maternal education, maternal ppBMI, and GDM) and polygenic risk scores for obesity (adjusted for ethnicity and sex) with the trajectory classification. Analysis of covariance was used to evaluate differences in continuous cardiometabolic outcomes and body fat depots at age 6 years, and eating behaviors at age 4.5y, between the trajectory classes (adjusted for sex, parity, ethnicity, maternal education, ppBMI, GDM, and GWG rate category). Binary logistic regression was used for dichotomous outcomes. The trajectory class with the largest number of participants was used as the reference group in all the above models. Complete case analyses (samples without missing covariates) for the above models are shown in the supplementary Tables S2 to S4.

## Results

### Growth trajectories from birth to 6 years of age

**Figure 1:**
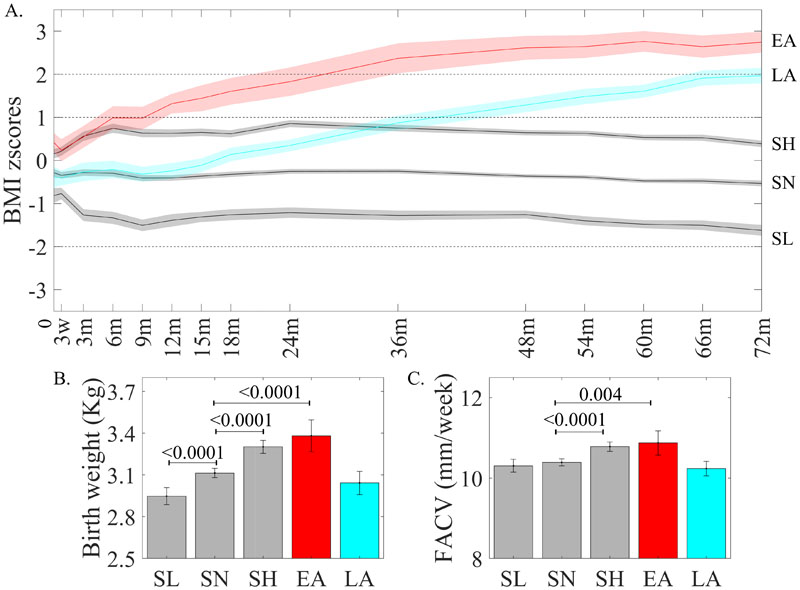
(A) Postnatal BMI trajectory classes. Differences in (B) Birthweight and (C) Fetal Abdominal Circumference Velocity (FACV) across BMI trajectory Classes Trajectory modeling revealed 5 distinct trajectory classes (N=994). The three stable trajectories, Stable Normal Low (SNL), N=131 (13.2%), Stable Normal (SN), N=458 (46.1%), Stable Normal High (SNH), N=264 (26.6%) stayed within the normal range of BMI (broken lines depict WHO thresholds for overweight (1SD), obesity (2SD) and thinness (-2SD) at age 6 years). The Early Acceleration (EA, N=58 (5.8%)) trajectory showed BMI acceleration immediately after birth and crossed obesity threshold after 2y of age. The Late Acceleration (LA, N=83 (8.4%)) trajectory was close the SN trajectory in the first year, started accelerating after age 1y and was close to the obesity threshold by age 6 years. Mean birth weights (Kg, N=994) of SNH and EA children were higher, and that of SNL children was lower than SN children. FACV (mm/week, N=927) between 2^nd^ and 3^rd^ trimester was higher in EA and SNH classes as compared to SN while LA and SNL classes have similar FACV to the SN class. Bonferroni corrected P-values are indicated.

Five growth trajectory patterns were identified (Figure 1). Three trajectory patterns were within the WHO BMI threshold for healthy BMI (-2 <BMI z-score < 1)^20^ and formed ~86% of the cohort. Since these three growth trajectory patterns remained stable and only differed in their levels, we classified them as Stable Normal Low (SNL,13.18%), Stable Normal (SN,48.08%) and Stable Normal High (SNH,26.56%). The remaining two trajectory patterns were characterized by BMI acceleration and a mean BMI at age 6 years above the “healthy range”. The early acceleration (EA,5.84%) trajectory showed BMI acceleration from birth, crossing the overweight threshold by age 1y, and the obesity threshold (+2SD) by age 2y. The late acceleration (LA,8.35%) trajectory was close to the SN trajectory in the first year of life but subsequently showed BMI acceleration. This trajectory crossed the overweight threshold at 3y of age and approached the obesity threshold by age 6 years. SNH and EA children had higher, while SNL children had lower birthweights compared to SN children (Fig. 1B). EA and SNH children had higher FACV than SN children, while the FACV of LA children were similar to that of SN and SNL children (Fig. 1C). Concordant with their higher FACV, EA children also had elevated abdominal fat volumes at birth (supplementary Table S5). The LCGMM analysis using cases without missing longitudinal BMI data also yielded a 5-class solution with similar trajectory patterns (supplementary Figure S3). The demographic and prenatal characteristics corresponding to the trajectory patterns are detailed in Table 1.

### Association of early life exposures with childhood growth trajectories

The mutually adjusted multinomial logistic regression analysis (Table 2) indicated ethnic differences in the growth trajectories. While Indian ethnicity was associated with both EA and LA trajectory classes, Malay ethnicity was uniquely associated only with the EA trajectory classes. High maternal ppBMI and shorter breastfeeding durations were common risk factors linked to both EA and LA trajectory classes. Nulliparity was uniquely linked to increased odds of having late BMI acceleration, while lower maternal educational attainment was uniquely linked to early BMI acceleration. Among maternal pregnancy complications, GDM and hypertensive disorders of pregnancy were not associated with any of the trajectories. However, excessive GWG was linked to increased odds of being in the SNH trajectory class. Shorter breastfeeding duration also increased the odds of being in the EA and LA trajectory classes.

### Association of genetic risk of obesity with childhood growth trajectories

After adjusting for ethnicity and sex, higher offspring polygenic risk scores for obesity were found to be associated with increased odds of being in the EA (~3 fold higher) and LA (~2 fold higher) trajectory classes and lower odds of being in the SNL (~2 fold lower) trajectory class, relative to the SN trajectory class.

### Association of childhood growth trajectories with eating behaviors at 4.5y

Comparison of the objectively measured eating behavioral traits from an *ad libitum* meal at age 4.5y across the trajectory classes, indicated that LA children had an elevated eating rate, with fewer chews per gram consumed, as well as higher energy intake (both solids and total intake) when compared to SN children (Table 3, unadjusted mean±sd shown in supplementary Table S6, complete case analysis shown in supplementary Table S3). The elevation in energy intake in EA children relative to SN children was comparable to that of LA children, despite EA children not exhibiting any altered eating behavior traits.

### Association of childhood growth trajectories with cardio-metabolic measures, ectopic fat and abdominal fat compartments

Associations of the trajectories with fat depots and cardiometabolic outcomes are shown in Table 4. Both EA and LA patterns were linked to increased odds of prehypertension/hypertension, elevated fasting insulin, and elevated HOMA-IR. However, fasting glucose was elevated only in LA children. SAT volumes were higher in EA, LA and SNH children and lower in SNL children. IAT volumes and liver fat were higher in EA and LA children, while IMCL did not vary across the trajectory classes. Similar findings were observed when complete case analyses were performed (supplementary Table S4)

### Comparison the determinant of SNH and LA trajectories

The SNH trajectory pattern was characterized by elevated BMI which was stable between 0 to 6 years. The LA trajectory pattern had stable normal BMI in infancy, but growth acceleration after infancy. To understand the determinants of the shift in growth trajectory patterns, we also compared the associations of the LA trajectory relative to the SNH trajectory with intrauterine growth (supplementary Table S7), early life determinants (supplementary Table S8) and eating behaviors (supplementary Table S9). LA children had lower FACV and birthweight than SNH children, despite having a higher genetic risk of obesity, which was suggestive of intrauterine growth constraints. Indian ethnicity and nulliparity were linked to a nearly 4-fold higher and 2-fold higher odds, respectively, of being in the LA trajectory. LA children also had reduced chews/g relative to SNH children.

## Discussion

The current study adds to a growing body of work using LCGMM to identify heterogeneous growth patterns in different populations^22–25^. In a multi-ethnic Asian cohort, we identified 5 BMI z-score trajectory patterns in the first six years of life. Three trajectories remained stable within the healthy BMI range while the remaining two showed BMI acceleration (~14% of the cohort). The EA trajectory showed BMI acceleration immediately after birth, crossing the obesity threshold as early as 2y, while the LA trajectory showed acceleration only after infancy and approached the obesity threshold by age 6 years. In utero, EA children had the highest FACV, while that of LA children had the lowest FACV. Of note, although the current study was conducted in an Asian cohort in the setting of a developed, high-income country, LCGMM has uncovered similar stable and accelerating BMI trajectory patterns in other contemporary cohorts with very different racial/ethnic and socioeconomic profiles^23–25^.

Children in the EA and LA trajectory classes showed evidence of early cardiometabolic alterations concordant with LCGMM-derived accelerating BMI trajectories in earlier studies^22, 25^. Liver fat, subcutaneous and intra-abdominal adipose tissue, odds of prehypertension/hypertension and HOMA-IR were elevated in both groups. While fasting insulin and HOMA-IR were highest in EA children, only LA children had elevated fasting glucose levels. The elevation of fasting glucose in LA children could be a result of multiple factors: increased resistance to action of insulin, inadequate beta cell function/mass, increased substrate availability for gluconeogenesis, increased glycogenolysis, and/or increased glucagon secretion. The precise underlying mechanisms are outside the scope of the present study. Abdominal and liver fat accumulation have been previously linked to increased cardiometabolic risk in pre-pubertal children^26, 27^. Thus, tracking of these cardiometabolic and body fat partitioning phenotypes to adolescence and adulthood may be a potential mechanism through which BMI acceleration in infancy and early childhood influences long-term cardiometabolic health. Retrospective analyses of growth trajectories in adults with coronary heart disease or type 2 diabetes have showed that these individuals were born with lower birth weights, had poor growth in infancy and BMI acceleration in early childhood^1–3^. While this trajectory pattern resembles the LA trajectory pattern, there is one major difference. BMI acceleration was linked to lower birth weight in these retrospective studies. In the current study, birthweights and FACV of the LA group were not different from the SN group, while the prevalence of SGA was slightly higher (16.9% vs 11.4%). Potential explanations for this could be that some of the older developmental cohorts experienced periods of nutritional stress before or during pregnancy, included many women of low BMI or were located in developing countries^1–3, 28^. We also found that EA group showed had elevated FACV, birthweights and neonatal abdominal fat volumes, when compared to SN children. Thus, the early growth acceleration in EA children doesn’t seem to be a compensatory catch-up response to constrained intrauterine growth^29^. The obesogenic trajectory patterns we identified may be reflective of children growing up in contemporary well-nourished populations. Similar trends have been observed with prospective LCGMM analysis in other populations. For instance, in an American cohort, early BMI acceleration was associated with higher birthweights while children showing late BMI acceleration had comparable birthweights to that of children having a normal trajectory^23^. In an Ethiopian cohort, children with early or late BMI acceleration had mean birthweights which were comparable to that of children in the stable trajectories^25^.

Indian ethnicity was a common risk factor for both EA and LA trajectory patterns, while Malay ethnicity was uniquely linked to the EA trajectory pattern. The associations of the minority ethnic groups (Indians and Malay) with obesogenic growth trends during early childhood was noteworthy, given the fact that prevalence of adulthood obesity, type 2 diabetes as well as coronary heart disease related morbidity and mortality are higher in Indians and Malays, relative to Chinese in this population^30–32^. Independent of ethnicity, lower maternal educational attainment was uniquely linked to the EA trajectory pattern. Minority ethnicity and low maternal educational attainment may be proxies for several unmeasured vulnerabilities. These may include both socio-structural vulnerabilities like unequal access to education, network inequalities, access to social capital, social mobility and socio-structural barriers to healthy/active living^33, 34^, as well as psychosocial vulnerabilities like chronic stress and cognitive adaptations secondary to bias or discrimination and affective states linked to lower subjective socioeconomic status^35–37^. Modifying the social and environmental contexts which give rise to such vulnerabilities may require interventions at the level of social and governmental policies^38, 39^. It is notable that Indian ethnicity was associated with both EA and LA trajectory patterns. Indian infants have been reported to have lower birthweight compared to infants from other ethnicities (nearly 280-350g lower than Caucasian infants^40^). Within the GUSTO cohort, the differences in birthweight between Indian infants and Chinese/Malay infants were less pronounced (66 g lower than Malay infants (P = 0.096), and 75g lower than Chinese infants (P = 0.028)). While it has been speculated that poor nutrition and infection load, before and during pregnancy may contribute to this phenomenon, the lower birthweight has been found to persist even in second generation Indian infants born in developed countries^41^. Interestingly, we observed that the situation was reversed by age 6 years, when Indian children were 1.36 kg heavier than Malay children (P = 0.014) and 1.84 kg heavier than Chinese children (P <0.001)). These findings suggest that they experienced higher growth acceleration between ages 0 to 6 years, and are consistent with the associations of Indian ethnicity with the EA and LA trajectory patterns. The nature of the underlying factors constraining intrauterine growth and causing accelerated growth in infancy/childhood in Indians remain to be fully elucidated.

Obesity PRS was a common non-modifiable risk factor for both EA and LA trajectory patterns. The risk of the EA trajectory pattern linked to obesity PRS was nearly double that of the LA trajectory pattern, suggesting a higher genetic contribution to the EA trajectory pattern. EA children had the highest FACV which was concordant with their high obesity PRS. Despite having an elevated obesity PRS, LA children had the lowest FACV among all the trajectory classes. It is plausible that LA children experienced an intrauterine growth constraint that does not allow them to grow to their true genetic growth potential. Interestingly, nulliparity at study recruitment was another non-modifiable risk factor uniquely linked with the LA trajectory pattern. Nulliparity has been postulated to present a maternal physiological constraint that can limit fetal growth to levels below its genetic growth potential, possibly due to inadequate remodeling of the spiral artery to increase utero-placental blood flow or because of a smaller uterus size^42^. The absence of such a constraint in postnatal life may create a discordant postnatal growth pattern, when children with a genetic predisposition for obesity are born to nulliparous mothers.

High ppBMI was the only antenatal risk factor linked to both EA and LA trajectory patterns. Concordant with the known protective effects of breastfeeding against increased weight gain in infancy^43^, we found shorter duration of breastfeeding to be linked to both EA and LA trajectory patterns. Both high maternal ppBMI and shorter breastfeeding duration have also been identified as risk factors for accelerating BMI trajectory patterns in other populations^23, 24^.

We found LA children to have elevated energy intake in an *ad libitum* meal. LA also displayed an obesogenic eating style with a faster eating rate and reduced chews per gram. These traits have been previously linked with childhood adiposity^15, 44^. Given that the models included parity as a covariate, these associations seem to be independent of the faster eating rates linked to first-born children^45^. These traits are modifiable through better parental feeding practices, parental awareness of child appetite and food-cue responsiveness, and through changes to the child’s early food environment^46–48^.

In the additional analysis we carried out comparing LA children to SNH children, we found Indian ethnicity and nulliparity to increase the odds of having a LA trajectory pattern (relative to SNH pattern). LA children also had lower chews/g than SNH children. These factors may contribute to the intrauterine and postnatal growth patterns observed in LA children (lowest FACV despite having an elevated obesity PRS, and growth acceleration after infancy).

**Figure 2:**
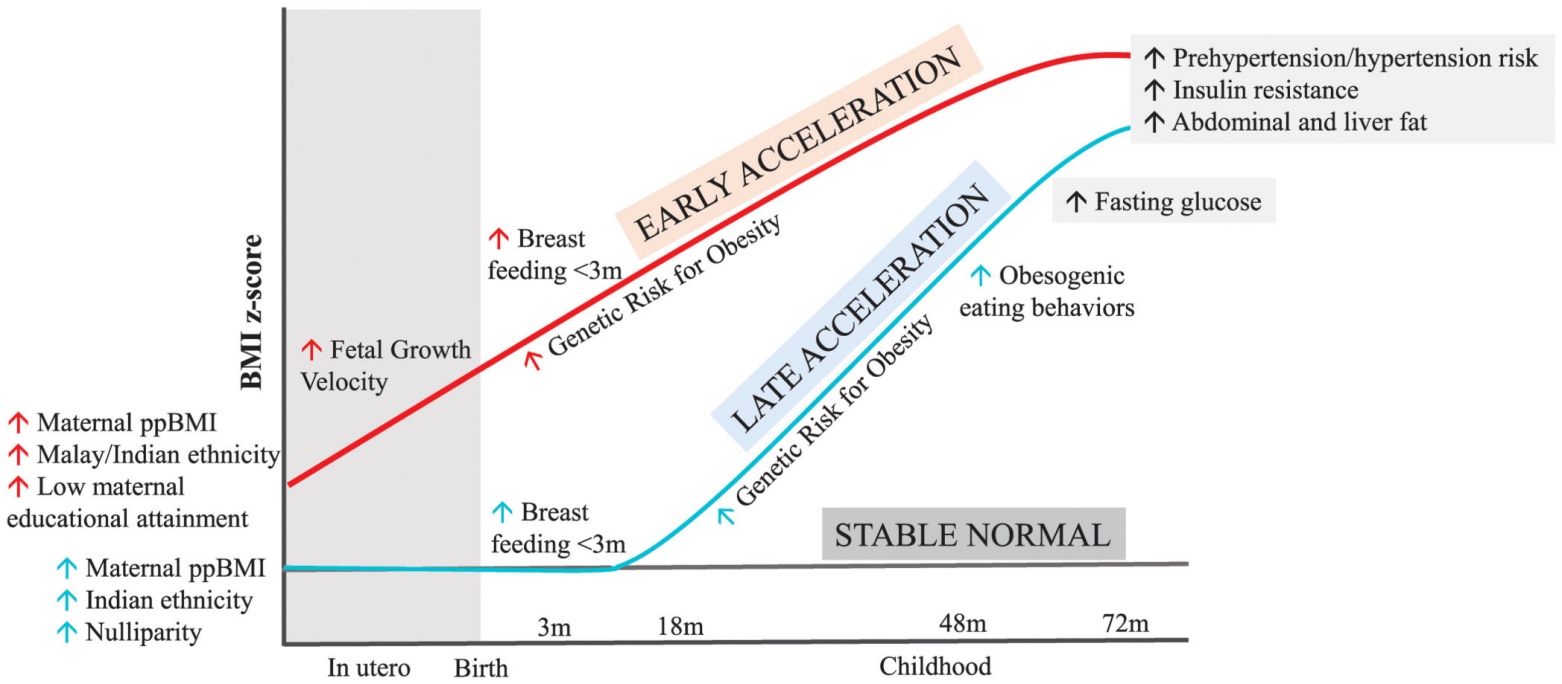
Schematic diagram showing pre- and post-natal risk factors and postnatal behavioral and cardiometabolic assessments associated with early acceleration (red color) and late acceleration (blue color) trajectory patterns. Up-arrow indicates an increase in risk of being classified into a trajectory class linked to risk factor, or an elevated outcome relative to the stable normal trajectory pattern.

A key strength of the current study is that we could leverage data from a deeply phenotyped prospective Asian mother-offspring cohort to identify obesogenic growth patterns linked with metabolic alterations during childhood and identify the related risk factors (A conceptual schematic showing the unique and shared risk factors linked to these patterns is shown in Fig. 2). In particular, the data on antenatal risk factors, ultrasound-based FACV, obesity PRS, objectively measured eating behaviors and imaging-based body abdominal and ectopic fat assessments have not been commonly simultaneously available in earlier studies on growth trajectory modeling. The ethnic groups included in the study are representative of nearly half of the global population who live in regions where the economic and social burden of obesity-related comorbidities is high.

Several study limitations should be noted. A limitation of LCGMM is that membership to a trajectory class can only be assigned retrospectively^49^. Despite this limitation, the determinants of the obesogenic trajectory patterns provide opportunities for prospective identification of high-risk children. The EA trajectory group had a relatively small sample size (<6%), hence the reported associations should be interpreted with caution. Replication studies in larger cohorts is warranted. We might be underpowered to detect associations with some of the weaker (but biologically important) determinants. Group-based trajectory modeling masks individual growth milestones like timing of infancy BMI peak or adiposity rebound which have also been linked to increased cardiometabolic risk^49^. Cardiometabolic, imaging and eating behavior assessments were available only in subsets of the cohort, which could have induced selection bias and limited the power to detect differences across the classes. Maternal pre-pregnancy weights were self-reported, which could have resulted in an under-reporting of their weights. Fetal biometry assessments using ultrasound are highly operator dependent. We were not able to account for paternal influences on the growth patterns in the current work.

In conclusion, our analysis of the BMI z-scores of a multi-ethnic Asian cohort from a developed nation identified temporal heterogeneity in the onset of obesogenic growth patterns, with one already apparent *in utero* and the one becoming apparent after infancy. Both these patterns were linked to early alterations in cardiometabolic risk markers and were associated with both shared as well as unique risk factors. These risk factors are relevant for early risk stratification and focusing on appropriate developmental windows for effective intervention. The underlying factors driving obesogenic growth patterns may vary among populations that inhabit different sociodemographic and economic settings and warrant an in-depth understanding for the development of new approaches in early life to mitigate risk of obesity. Population-centric analysis of these growth patterns can inform the design of localized prospective studies for testing out specific interventions and building precision into the global efforts to minimize the burden of obesity and related comorbidities.

### Ethics approval

This study was approved by both the National Healthcare Group Domain Specific Review Board (D/2009/021 & B/2014/00411) and the SingHealth Centralized Institutional Review Board (2018/2767 & 2019/2406), and has been performed in accordance with the ethical standards laid down in the 1964 Declaration of Helsinki and its later amendments Written informed consent was obtained from all children and their parents.

## Supplementary Material

Supplementary material

## Figures and Tables

**Table 1:** Maternal, pregnancy, offspring and postnatal characteristics across the trajectory classes.

	N	SNL	SN	SNH	EA	LA
**Maternal characteristics**						
Ethnicity	994					
*Chinese*		68(51.9)	295(64.4)	149(56.4)	16(27.6)	35(42.2)
*Malay*		24(18.3)	98(21.4)	81(30.7)	28(48.3)	18(21.7)
*Indian*		39(29.9)	65(14.2)	34(12.9)	14(24.1)	30(36.1)
Maternal education	981					
*Below Secondary*		41(31.8)	122(27.0)	74(28.5)	26(44.8)	23(28.0)
*Post-Secondary*		42(32.6)	154(34.1)	95(36.5)	22(37.9)	29(35.4)
*University*		46(35.7)	176(38.9)	91(35.0)	10(17.2)	30(36.6)
Household income per month (S$)	925					
*≤1999*		15(12.3)	57(13.4)	31(12.8)	10(17.9)	14(17.9)
*2000-5999*		79(64.9)	217(50.9)	133(54.7)	40(71.4)	42(53.8)
*≥6000*		28(23.0)	152(35.7)	79(32.5)	6(10.7)	22(28.2)
Maternal age (at recruitment)	994	30.59±5.20	31.00±4.95	30.60±5.21	30.77±4.92	30.25±5.07
Pre-pregnancy BMI	910	22.21±4.64	21.95±3.83	23.17±4.50	25.53±4.97	24.17±4.97
Maternal height (cm)	975	156.84±5.44	158.84±5.54	158.16±5.54	158.90±.5.85	158.00±6.05
Paternal BMI	669	25.3±4.6	25.2±4.2	26.2±4.3	27.7±3.8	26.9±4.8
Parity	994					
*Nulliparous*		47 (35.9)	208 (45.4)	125 (47.3)	25 (43.1)	46 (55.4)
*Parous*		84 (64.1)	250 (54.6)	139(52.7)	33(56.9)	37(44.6)
**Pregnancy characteristics**						
OGTT 26th week						
*Fasting Glucose (mmol/L)*	950	4.30±0.39	4.30±0.44	4.37±0.42	4.48±0.63	4.45±0.72
*2-hour Glucose (mmol/L)*		6.47±1.52	6.48±1.39	6.47±1.46	6.84±1.46	6.64±1.92
Gestational diabetes mellitus	950					
*No*		102(80.3)	361(83.0)	210(83.0)	46(80.7)	60(76.9)
*Yes*		25(19.7)	74(17.0)	43(17.0)	11(19.3)	18(23.1)
Rate of gestational weight gain *	877					
*Inadequate*		17(14.8)	59(14.4)	28(12.2)	4(7.8)	11(15.1)
*Adequate*		55(47.8)	165(40.3)	68(29.7)	14(27.5)	19(26.0)
*Excessive*		43(37.4)	185(45.2)	133(58.1)	33(64.7)	43(58.9)
Smoking (during)	982					
*No*		127(97.7)	443(97.8)	255(97.3)	56(96.6)	80(100)
*Yes*		3(2.3)	10(2.2)	7(2.7)	2(3.4)	0(0)
Hypertensive disorders of pregnancy	993					
*No*		123(94.6)	430(93.9)	253(95.8)	56(96.6)	76(91.6)
*Yes*		7(5.4)	28(6.1)	11(4.2)	2(3.4)	7(8.4)
Gestational Age (weeks)	994	38.90±0.99	39.03±0.96	39.10±1.03	38.84±1.00	38.86±1.07
**Offspring Characteristics**						
Sex (%Males)	994	54.2	50.4	51.9	60.3	57.8
Size for gestational age	994					
*Appropriate for GA*		99(75.6)	347(75.8)	187(70.8)	40(69.0)	61(73.5)
*Small for GA*		25(19.1)	52(11.4)	9(3.4)	0(0.00)	14(16.9)
*Large for GA*		7(5.3)	59(12.9)	68(25.8)	18(31.0)	8(9.6)
Polygenic Risk Score (Obesity)	939	-0.17±0.50	-0.04±0.51	0.03±0.49	0.25±0.53	0.11±0.42
**Postnatal Nutrition**						
Duration of Breastfeeding	994					
*<3m*		55(42.0)	174(38.0)	106(40.2)	36(62.1)	39(47.0)
*≥ 3m*		76(58.0)	284(62.0)	158(59.8)	22(37.9)	44(53.0)

Categorical variables are presented as number (percentage) in each category and the continuous variables are represented by mean (standard deviation). SNL: Stable Normal Low, SN: Stable Normal, SNH: Stable Normal High, EA: Early Acceleration, LA: Late Acceleration. GA: Gestational Age. BMI: Body Mass Index. OGTT: Oral Glucose Tolerance Test.

* Calculated between 15-35 weeks

**Table 2:** Multinomial logistic regression models for evaluating the odds of being classified in a specific trajectory class due to early life predictors.

Exposures	Trajectory Classification			
	SNL vs. SN	SNH vs. SN	EA vs. SN	LA vs. SN
**Model-1: Influence of maternal antenatal factors (mutually adjusted)**				
Ethnicity				
*Malay vs. Chinese*	0.85[0.48,1.52]	1.27[0.84,1.93]	3.22[1.49,6.93]	1.07[0.53, 2.18]
	*0.592*	*0.256*	***0.003***	*0.845*
*Indian vs. Chinese*	2.30[1.37,3.87]	0.84[0.51,1.37]	3.36[1.47,7.69]	3.10[1.68,5.74]
	***0.002***	*0.480*	***0.004***	*<**0.0001***
Maternal Education				
*Secondary vs. University*	1.32[0.78,2.25]	0.95[0.62,1.46]	2.70[1.13,6.44]	1.22[0.63,2.38]
	*0.306*	*0.818*	***0.025***	*0.553*
*Post-secondary vs. University*	1.13[0.78,2.25]	0.98[0.66,1.44]	1.80[0.77,4.19]	1.03[0.56,1.89]
	*0.646*	*0.903*	*0.172*	*0.929*
Parity				
*Nulliparous vs. Parous*	0.72[0.46,1.13]	1.13[0.81,1.59]	1.46[0.78,2.75]	1.85[1.09,3.16]
	*0.152*	*0.480*	*0.241*	***0.023***
Maternal Age (years)	0.98[0.94,1.02]	0.99[0.96,1.03]	1.03[0.97,1.09]	0.99[0.94,1.05]
	*0.318*	*0.576*	*0.332*	*0.748*
ppBMI (kg/m^2^)	1.00[0.94,1.06]	1.06[1.01,1.11]	1.12[1.05,1.20]	1.10[1.03,1.17]
	*0.966*	***0.012***	***0.001***	***0.005***
Maternal height (cm)	0.95[0.91,0.98]	0.98[0.95,1.01]	1.03 [0.98,1.09]	0.98[0.94,1.03]
	***0.003***	*0.157*	*0.247*	*0.408*
Gestational diabetes mellitus				
*Yes vs. No*	1.19[0.69,2.06]	1.08[0.69,1.69]	1.35[0.61,2.98]	1.33[0.69,2.55]
	*0.535*	*0.741*	*0.458*	*0.396*
Hypertensive disorders of pregnancy				
*Yes vs. No*	0.99[0.41,2.40]	0.50[0.24,1.05]	0.27[0.06,1.28]	0.93[0.37,2.35]
	*0.978*	*0.066*	*0.100*	*0.879*
Rate of Gestational Weight Gain				
*Inadequate vs. Adequate*	0.75[0.39,1.43]	1.16[0.66,2.01]	0.68[0.20,2.26]	1.54[0.67,3.53]
	*0.379*	*0.610*	*0.530*	*0.308*
*Excessive vs. Adequate*	0.72[0.44,1.18]	1.52[1.03,2.25]	1.24[0.60,2.53]	1.50[0.78,2.73]
	*0.187*	***0.036***	*0.561*	*0.239*
**Model -2: Influence of offspring polygenic risk score for obesity (adjusted for ethnicity & sex)**				
Polygenic Risk Scores (Obesity)	0.60[0.40,0.91]	1.34[0.98,1.83]	3.49[1.90,6.40]	1.88[1.13,3.14]
	***0.015***	*0.069*	*<**0.0001***	***0.015***
**Model-3: Influence of breastfeeding duration (adjusted for ethnicity, ppBMI, education & gestational diabetes)**				
Breastfeeding duration	1.05[0.68,1.63]	1.00[0.70,1.39]	1.74[0.94,3.22]	1.30[0.76,2.20]
*<3m Vs ≥3 months*	*0.825*	*0.945*	0.079	0.36

Odds ratio [95%CI] with respect to the reference trajectory (stable normal) are presented. P values are indicated below each reported odds ratio. P values <0.05 are highlighted in bold. SNL: Stable Normal Low, SN: Stable Normal, SNH: Stable Normal High, EA: Early Acceleration, LA: Late Acceleration. BMP: Body Mass Index, ppBMI: Pre-pregnancy Body Mass Index.

**Table 3:** Adjusted mean differences (β [95%CI]) in eating behaviors measured at age 4.5y across the trajectory classes.

Behavioral Assessments	SNL Vs SN	SNH Vs SN	EA Vs SN	LA Vs SN
**Eating behavior at age 4.5y**				
Eating Rate (g/min)	-0.78[-1.93, 0.37]	0.54[-0.37, 1.45]	0.59[-1.07, 2.26]	1.37[-0.12, 2.86]
	*0.184*	*0.241*	*0.483*	0.072
Chews per g	0.68[-1.80, 3.16]	-0.62[-2.58, 1.34]	-0.42[-4.00,3.16]	-4.15[-7.36, -0.93]
	*0.592*	*0.538*	*0.818*	***0.011***
Oral Exposure per bite (s)	1.44[-2.73, 5.61]	1.35[-1.95, 4.65]	-1.91 [-7.93, 4.11]	2.19[-3.21, 7.60]
	*0.498*	*0.423*	*0.534*	*0.427*
Bite size (g/bite)	-0.13[-0.57, 0.31]	0.19[-0.15, 0.54]	-0.05[-0.68, 0.59]	0.52[-0.05, 1.09]
	*0.551*	*0.273*	*0.879*	*0.073*
Energy Intake, solids (kcal)	-21.01[-67.65, 25.64]	13.77[-23.14,50.68]	55.61[-11.87,123.09]	59.16[-1.38, 119.70]
	*0.377*	*0.465*	*0.106*	0.055
Energy Intake, total (kcal)	-35.32[-87.69, 17.05]	20.40[-21.05, 61.86]	62.72[-13.06, 138.50]	67.73[-0.25, 135.70]
	*0.186*	*0.335*	*0.105*	0.051
Eating in Absence of Hunger (kcal)	9.62[-6.34, 25.57]	6.61[-5.62, 18.85]	-5.01 [-27.18, 17.17]	11.58[-8.78, 31.93]
	*0.238*	*0.290*	*0.658*	*0.265*

Models were adjusted for ethnicity, pre-pregnancy body mass index, sex, maternal education, rate of gestational weight gain, gestational diabetes, and parity. The stable normal trajectory pattern was used as the reference group for all models. Each row represents a separate model. P values are reported below each effect size measure. P values <0.05 are highlighted in bold. SNL: Stable Normal Low, SN: Stable Normal, SNH: Stable Normal High, EA: Early Acceleration, LA: Late Acceleration.

**Table 4:** Adjusted mean differences (β [95%CI]) in body fat depots and cardiometabolic assessments at age 6 years across the trajectory classes (Adjusted odds ratio [95%CI] shown for dichotomous outcomes)

Cardiometabolic Assessments	SNL Vs SN	SNH Vs SN	EA Vs SN	LA Vs SN
**Fat depots at age 6 years**				
SAT (cm^3^)	-214.82[-341.16, -88.48]	132.37[42.64,222.10]	1690.10[1534.06,1846.13]	1076.32[931.33,1221.32]
	***0.001***	***0.004***	*<**0.0001***	*<**0.0001***
VAT (cm^3^)	-3.24[-28.14, 21.66]	10.58[-7.12,28.28]	246.49[215.71,277.27]	158.39[129.79,187.00]
	*0.799*	*0.241*	*<**0.0001***	*<**0.0001***
Liver Fat	0.07[-0.28,0.40]	0.07[-0.17,0.31]	1.30[0.88,1.73]	0.74[0.36,1.12]
(% weight)	*0.733*	*0.566*	*<**0.0001***	*<**0.0001***
IMCL	-0.02[-0.26,0.21]	-0.01 [-0.17,0.15]	0.09[-0.19,0.37]	-0.02[-0.27,0.24]
(% water signal)	*0.850*	*0.904*	*0.535*	*0.899*
**Cardio-metabolic outcome at age 6**				
Prehypertension/Hypertension*	0.52[0.22,1.22]	1.37[0.82,2.31]	2.29[0.99,5.30]	2.15[1.07,4.31]
	*0.133*	*0.231*	0.052	***0.031***
Fasting Glucose (mmol/l)	-0.07[-0.17,0.02]	0.05[-0.03,0.13]	0.05[-0.10,0.20]	0.15[0.03,0.27]
	*0.140*	*0.206*	*0.520*	***0.017***
Fasting Insulin (mU/l)	-0.81[-1.66,0.05]	-0.02[-0.71,0.67]	4.10[2.80,5.40]	3.08[1.98,4.18]
	*0.065*	*0.958*	*<**0.0001***	*<**0.0001***
HOMA-IR	-0.17[-0.35,0.03]	0.04[-0.11,0.20]	1.09[0.80,1.38]	0.63[0.38,0.88]
	*0.088*	*0.590*	*<**0.0001***	*<**0.0001***

All models were adjusted for ethnicity, pre-pregnancy body mass index, sex, maternal education, rate of gestational weight gain, gestational diabetes, and parity. The stable normal trajectory pattern was used as the reference group for all models. Each row represents a separate model. P values are reported below each effect size measure. P values <0.05 are highlighted in bold. SNL: Stable Normal Low, SN: Stable Normal, SNH: Stable Normal High, EA: Early Acceleration, LA: Late Acceleration, SAT: Subcutaneous Adipose Tissue, IAT: Intra-abdominal Adipose Tissue, IMCL: Intramyocellular Lipids. HOMA-IR: Homeostatic Model Assessment for Insulin Resistance.

* Values indicate odds ratio from binary logistic regression due to dichotomous outcome.

## Data Availability

The data used in the manuscript is available on request, on approval by the GUSTO executive committee.

## Key Messages

- In an Asian multi-ethnic prospective mother-offspring cohort (Indian, Chinese and Malay ethnicities), we identified two distinct obesogenic body mass index (BMI) z-score trajectory patterns in early childhood, which were both linked to early cardiometabolic alterations.
- The early acceleration trajectory pattern showed evidence for high fetal abdominal circumference growth velocity, growth acceleration immediately after birth, and crossed the obesity threshold by age 2y, while the late acceleration trajectory pattern showed growth acceleration after infancy and approached the obesity threshold by age 6 years.
- Indian ethnicity, high pre-pregnancy BMI, high polygenic risk scores for obesity and shorter breastfeeding duration were common risk factors for both groups.
- Malay ethnicity and low maternal educational attainment were uniquely associated with early acceleration, while nulliparity and obesogenic eating behaviors in early childhood were uniquely associated with late acceleration.
- The determinants of the obesogenic trajectory patterns may be useful for developing early risk stratification and intervention approaches to counteract metabolic adversities linked to childhood obesity.
