# Supplementary material for: Longitudinal characterization of determinants associated with obesogenic growth patterns in early childhood
Source: Int J Epidemiol. Author manuscript; Available in PMC 2023 Apr 20. (PMC10114026; doi:10.1093/ije/dyac177)
Supplement: Supplementary material [file EMS163493-supplement-Supplementary_material.docx]

**SUPPLEMENTARY MATERIALS**

Figure S1. Recruitment flowchart and sample availability for growth, cardiometabolic and behavioral assessments.

Recruitment **1450**

Term singletons with homogeneous parental ethnicity and at least four postnatal BMI measurement available between ages 0-6y

**1083**

Number of subjects at each time point used to estimate Body Mass Index with both weight and height/length measurements. For deriving trajectory classes, data from 994 children with at least 4 BMI measurements (including birth).

|  | Birth | 3w | 3m | 6m | 9m | 12m | 15m | 18m | 24m | 36m | 48m | 54m | 60m | 66m | 72m |
| --- | --- | --- | --- | --- | --- | --- | --- | --- | --- | --- | --- | --- | --- | --- | --- |
| BMI | 994 | 940 | 940 | 912 | 872 | 891 | 877 | 798 | 827 | 856 | 799 | 831 | 807 | 798 | 771 |
| Weight | 994 | 943 | 940 | 912 | 872 | 892 | 898 | 849 | 860 | 865 | 806 | 834 | 813 | 803 | 777 |
| Height/Length | 994 | 940 | 940 | 916 | 873 | 893 | 878 | 803 | 830 | 861 | 803 | 835 | 813 | 804 | 775 |

Fetal Abdominal Circumference Velocity (927)

MRI measures SAT, IAT at early infancy (≤21 days after birth) (312)

Eating Behavior (285), Eating in absence of hunger (290) at 4.5 years

Prehypertension at 6 years (644)

Number of subjects with imaging, cardiometabolic and behavioral assessments

Fasting plasma insulin at 6 years (383)

Fasting plasma glucose at 6 years (485)

MRI measures SAT, VAT at 6 years (406)

MRS Liver Fat at 6 years (378)

MRS Intramyocellular Lipids (Soleus muscle) at 6 years (375)

**BMI assessments and Latent Class Growth Mixture Modelling of BMI z-score trajectories**

Serial weight and height/length measurements were made at in duplicate at birth, 3 weeks, 3m, 6m, 9m, 12m, 15m, 18m, 24m, 36m, 48m, 54m, 60m, 66m & 72m. Calibrated weighing scales were used for measuring weight (SECA 334 up to 18m and SECA 803 weighing scale beyond 18m). Recumbent length (SECA 210 mobile measuring mat) was to compute BMI until 24m, while standing height (SECA 213 Portable Stadiometer) was used for computing BMI beyond 24m. BMI measurements. BMI measurements were converted to age and sex standardized z-scores based on the 2006 World Health Organization (WHO) Child Growth Standards using the WHO Anthro macro for SPSS (v3.2.2, Jan 2011) (1). Latent class growth mixture modelling (LCGMM) was used to derive heterogeneous groups of BMI z-score trajectories using Mplus Version 8 (2), in 994 children who had at least 4 BMI datapoints (including birth). Quadratic polynomials were invoked in trajectory derivations to allow curved developmental patterns and default full information maximum likelihood analysis was used to handle missing data. Statistical criteria used for deriving the optimal number of trajectory classes are shown in Supplementary Table E1 (3, 4). The individual trajectories within each class are presented in Supplementary Figure E2. As a sensitivity analysis, LCGMM analysis was repeated using cases without any missing longitudinal BMI data (n=391) (Supplementary Figure E3).

Table S1. Statistical parameters used for deriving the optimum number of trajectories using Latent Class Growth Mixture Modelling. The optimal solution was derived by employing Bayesian information criterion (BIC), bootstrapped log-likelihood ratio test (p-value≤0.05), Vuong-Lo-Mendell-Rubin likelihood ratio test (p-value≤0.05), high entropy for classification (>0.80), high posterior probability of belonging to a class (≥0.70), and a membership in the smallest trajectory class of at least 5%.

| **Number of Trajectories** | **Bootstrapped**  **Log-likelihood Ratio Test** | **Adjusted Bayesian Information**  **Criteria** | **Vuong Lo**  **Mendel Rubin**  **test** | **Entropy** | **Average Posterior Probability of each trajectory class** | **Minimum percentage membership**  **in a class** |
| --- | --- | --- | --- | --- | --- | --- |
| 2 | <0.0001 | 33713.0 | <0.0001 | 0.90 | 0.97/0.97 | 40.2% |
| 3 | <0.0001 | 31885.6 | 0.028 | 0.90 | 0.96/0.96/0.95 | 10.9% |
| 4 | <0.0001 | 30546.6 | 0.064 | 0.90 | 0.97/0.94/0.94/0.94 | 8.1% |
| **5** | **<0.0001** | **29958.7** | **0.012** | **0.91** | **0.96/0.94/0.95/0.97/0.92** | **5.8%** |
| 6 | <0.0001 | 29384.1 | 0.46 | 0.88 | 0.90/0.95/0.97/0.93/0.94/0.90 | 4.1% |

Figure S2. Individual trajectories along with mean trajectory pattern of each LCGMM class are presented.


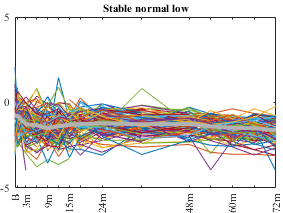

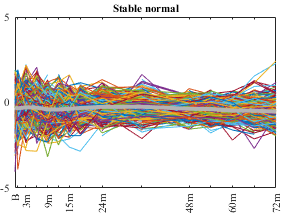


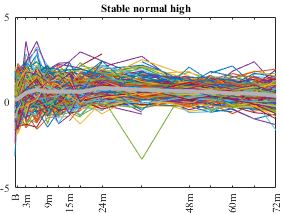

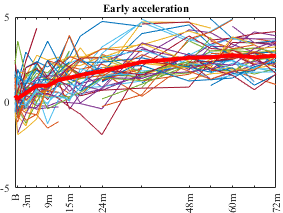


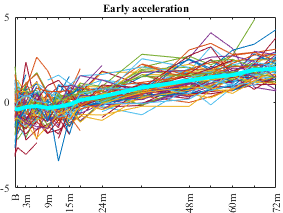


Figure S3. Trajectories derived from a subset of data with no missing values at any time point (391 samples) with SL (40), SN(169), SH(110), EA(27) and LA(45).


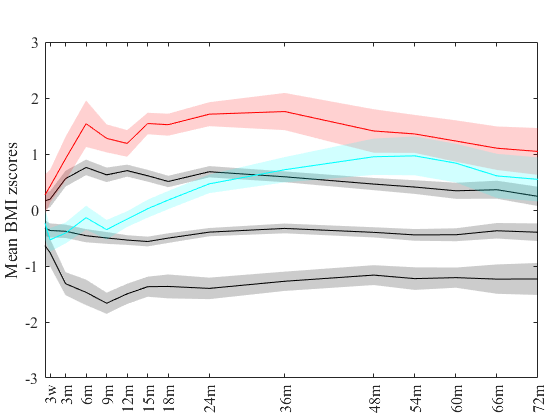


**Magnetic Resonance Spectroscopy Protocols**

All scans were performed without sedation using a Siemens Skyra 3T MR scanner. Single voxel proton magnetic resonance spectroscopy (MRS) was carried out to measure intramyocellular lipids (n=375) and liver fat (n=378). For liver fat, two 1cm^3^ voxels were taken at the left and right lobe of the liver using a point-resolved spectroscopy (PRESS) sequence without water suppression (TR/TR=30/2000ms, Averages=4). Respiratory gating was used to account for respiratory motion. LC-Model(5) was used to quantify area of the water resonance at 4.7 ppm and the lipid resonances between 0.5 and 3 ppm in the liver spectrum. T2 correction of the water and lipid peaks was carried out using T2 relaxation values reported previously in literature (6) and the corrected peak areas were used to estimate liver fat percentage by weight, according to previously validated methods (7, 8). A correction factor of 0.914 was used to correct for fact that the lipid peaks between 0.5-3ppm represent only 91.4% of the total lipid protons (9). In each voxel, the average liver fat was derived using the right and left lobe scans

For IMCL(10), a 1cm^3^ voxel was taken at the soleus muscle using PRESS sequence (with TR=2000ms, TE =33ms and Averages=24) following T1-weighted axial localization. LC-Model (5) was used to quantify the spectra. T2 correction of the water and lipid peaks was carried out using T2 relaxation values reported previously in literature (11) and IMCL as expressed as a percentage of the water peak area the water peak from a water unsuppressed scan in the same voxel.

**Supplementary References**

1. Organization WH**.** WHO child growth standards: length/height-for-age, weight-for-age, weight-for-length, weight-for-height and body mass index-for-age: methods and development. 2006.

2. Muthén L, Muthén B**.** Mplus. The comprehensive modelling program for applied researchers: user’s guide. 2015;5.

3. Jung T, Wickrama K**.** An introduction to latent class growth analysis and growth mixture modeling. Social and personality psychology compass. 2008;2(1):302-17.

4. Nylund KL, Asparouhov T, Muthén BO**.** Deciding on the number of classes in latent class analysis and growth mixture modeling: A Monte Carlo simulation study. Structural equation modeling. 2007;14(4):535-69.

5. Provencher SW**.** Estimation of metabolite concentrations from localized in vivo proton NMR spectra. Magnetic resonance in medicine. 1993;30(6):672-9.

6. Chabanova E, Bille DS, Thisted E, Holm J-C, Thomsen HS**.** MR spectroscopy of liver in overweight children and adolescents: Investigation of 1H T2 relaxation times at 3 T. European journal of radiology. 2012;81(5):811-4.

7. Longo R, Pollesello P, Ricci C, Masutti F, Kvam BJ, Bercich L, et al. Proton MR spectroscopy in quantitative in vivo determination of fat content in human liver steatosis. Journal of Magnetic Resonance Imaging. 1995;5(3):281-5.

8. Szczepaniak LS, Nurenberg P, Leonard D, Browning JD, Reingold JS, Grundy S, et al. Magnetic resonance spectroscopy to measure hepatic triglyceride content: prevalence of hepatic steatosis in the general population. American Journal of Physiology-Endocrinology and Metabolism. 2005.

9. Hamilton G, Yokoo T, Bydder M, Cruite I, Schroeder ME, Sirlin CB, et al. In vivo characterization of the liver fat 1H MR spectrum. NMR in biomedicine. 2011;24(7):784-90.

10. Michael N, Gupta V, Sadananthan SA, Sampathkumar A, Chen L, Pan H, et al. Determinants of intramyocellular lipid accumulation in early childhood. Int J Obes (Lond). 2020;44(5):1141-51.

11. Kautzky-Willer A, Krssak M, Winzer C, Pacini G, Tura A, Farhan S, et al. Increased intramyocellular lipid concentration identifies impaired glucose metabolism in women with previous gestational diabetes. Diabetes. 2003;52(2):244-51.

Table S2. Complete case analysis: Odds ratio [95%CI] characterizing the association of early life exposures on the trajectory classification with respect to the reference trajectory (stable normal) corresponding to complete data are presented. P values are indicated below each reported odds ratio. Exposures with significant effects have been highlighted in bold.

| Exposures | Trajectory Classification | | | | |
| --- | --- | --- | --- | --- | --- |
|  | **SL vs. SN** | | **SH vs. SN** | **EA vs. SN** | **LA vs. SN** |
| Influence of pre-natal environment factors (tested in the same model) | | | | | |
| Ethnicity | |  |  |  |  |
| *Malay vs. Chinese*  *Indian vs. Chinese* | | 1.07[0.57,2.02]  *0.839* | 1.38[0.87,2.18]  *0.177* | 4.10[1.78,9.46]  ***0.001*** | 1.22[0.57, 2.61]  *0.442* |
|  |  | 2.91[1.67,5.08]  ***<0.0001*** | 0.92[0.54,1.56]  *0.753* | 4.39[1.80,10.72]  ***0.001*** | 2.77[1.41,5.7]  ***0.003*** |
| Education | |  |  |  |  |
| *Secondary vs. University*  *Post-secondary vs. University* | | 1.43[0.81,2.54]  *0.219* | 1.06[0.66,1.69]  *0.807* | 2.94[1.16,7.45]  ***0.023*** | 1.28[0.63,2.62]  *0.498* |
|  |  | 1.19[0.69,2.03]  *0.534* | 0.99[0.65,1.51]  *0.957* | 1.96[0.81,4.78]  *0.137* | 0.95[0.49,1.84]  *0.885* |
| Parity | |  |  |  |  |
| *Nulliparous vs. Parous* | | 0.85[0.52,1.37]  *0.497* | 1.13[0.78,1.64]  *0.516* | 1.68[0.85,3.33]  *0.139* | 2.12[1.18,3.81]  ***0.013*** |
| Maternal Age (years) | | 1.00[0.95,1.05]  *0.878* | 1.00[0.96,1.04]  *0.828* | 1.04[0.98,1.11]  *0.212* | 1.00[0.94,1.06]  *0.952* |
| Pre-pregnancy BMI (kg/m^2^) | | 1.00[0.94,1.06]  *0.959* | 1.06[1.01,1.11]  ***0.019*** | 1.12[1.04,1.20]  ***0.001*** | 1.09[1.02,1.17]  ***0.007*** |
| Maternal height (cm) | | 0.94[0.90,0.98]  ***0.003*** | 0.97[0.94,1.01]  *0.096* | 1.02 [0.96,1.08]  *0.535* | 0.98[0.93,1.03]  *0.384* |
| Gestational diabetes mellitus | |  |  |  |  |
| *Yes vs. No* | | 1.25[0.71,2.19]  *0.438* | 1.09[0.69,1.74]  *0.705* | 1.39[0.62,3.12]  *0.421* | 1.35[0.69,2.63]  *0.378* |
| Hypertensive disorders of pregnancy | |  |  |  |  |
| *Yes vs. No* | | 1.03[0.42,2.55]  *0.944* | 0.53[0.24,1.16]  *0.114* | 0.32[0.07,1.54]  *0.157* | 0.92[0.35,2.44]  *0.863* |
| Rate of GWG | |  |  |  |  |
| *Inadequate vs. Adequate*  *Excessive vs. Adequate* | | 0.78[0.41,1.48]  *0.443* | 1.08[0.62,1.88]  *0.795* | 0.64[0.19,2.14]  *0.567* | 1.69[0.72,3.92]  *0.226* |
|  |  | 0.73[0.44,1.20]  *0.211* | 1.50[1.01,2.22]  ***0.044*** | 1.19[0.57,2.47]  *0.650* | 1.66[0.87,3.18]  *0.127* |
| Influence of Polygenic risk score for obesity (adjusted for ethnicity & gender) | | | | | |
| Polygenic Risk Scores (BMI) | | 0.60[0.39,0.90]  ***0.014*** | 1.34[0.98,1.84]  *0.068* | 3.48[1.89,6.43]  ***<0.0001*** | 1.86[1.12,3.09]  ***0.016*** |
| Influence of breastfeeding duration (adjusted for ethnicity, ppBMI, education & gestational diabetes mellitus) | | | | | |
| Breast feeding duration  *<3m Vs ≥3 months* | | 0.95[0.59,1.51]  *0.813* | 1.00[0.70,1.44]  *0.984* | 1.84[0.95,,3.55]  0.069 | 1.31[0.75,2.30]  *0.345* |

Table S3. Complete case analysis: Adjusted mean differences (β [95%CI]) in eating behavior, cardio-metabolic and body fat depot assessments across the trajectory classes. Odds ratio [95%CI] are shown for dichotomous outcomes. Comparisons are done with respect to stable normal trajectory P values are reported below each effect size measure.

| Outcome measures | SL Vs SN | SH Vs SN | EA Vs SN | LA Vs SN |
| --- | --- | --- | --- | --- |
| Eating behavior | | | | |
| Eating Rate (g/min) | -0.89[-2.07, 0.29]  *0.139* | 0.49[-0.45, 1.42]  *0.310* | 0.64[-1.18, 2.46]  *0.490* | 1.70[0.19, 3.21]  ***0.027*** |
| Chews/g | 0.71[-1.93, 3.36]  *0.597* | -0.58[-2.68, 1.51]  *0.585* | -1.42[-5.49, 2.66]  *0.495* | -4.56[-7.93, -1.19]  ***0.008*** |
| Oral Exposure per bite (s) | 1.22[-3.31 5.76]  *0.597* | 1.10[-2.49 4.70]  *0.547* | -2.68[-9.67, 4.31]  *0.452* | 1.22[-4.56, 7.00]  *0.679* |
| Bite size (g/bite) | -0.18[-0.62 0.26]  *0.417* | 0.11[-0.24, 0.45]  *0.539* | -0.08[-0.75, 0.59]  *0.813* | 0.63[0.07, 1.18]  ***0.027*** |
| Energy Intake, solids (kcal) | -24.26[-72.05,23.53]  *0.320* | 3.45[-34.42,41.32]  *0.858* | 34.28[-39.39, 107.96]  *0.362* | 63.01[2.07,123.95]  ***0.043*** |
| Energy Intake, total (kcal) | -42.31 [-96.05,11.42]  *0.123* | 10.91[-31.67, 53.49] *0.616* | 54.97[-27.87, 137.80]  *0.193* | 77.69[9.17, 146.20]  ***0.026*** |
| Eating in Absence of Hunger (kcal)^a^ | 8.89[-8.49, 26.26]  *0.316* | 4.87[-8.44, 18.18]  *0.473* | -5.78[-32.40, 20.83]  *0.670* | 9.72[-11.95, 31.40]  *0.379* |
| Covariates for adjusted models: sex, ethnicity, maternal education, ppBMI, rate of GWG (IOM), GDM, parity. ^a^Children with energy intake of less than 50 kcal in the eating in the absence of hunger assessment were excluded. | | | | |

Table S4. Complete case analysis: Adjusted mean differences (β [95%CI]) in cardio-metabolic and body fat depot at age 6y across the trajectory classes. Odds ratio [95%CI] are shown for dichotomous outcomes. Comparisons are done with respect to stable normal trajectory P values are reported below each effect size measure.

| Outcome measures | SL Vs SN | SH Vs SN | EA Vs SN | LA Vs SN | |  |
| --- | --- | --- | --- | --- | --- | --- |
| Cardio-metabolic outcome | | | | | |  |
| Prehypertension^*^  *Yes Vs No* | 0.54[0.22,1.31]  *0.173* | 1.32[0.75,2.32]  *0.340* | 2.09[0.83,5.29]  *0.118* | 2.16[1.02,4.56]  ***0.044*** | |  |
| Fasting Glucose mmol/l) | -0.10[-0.20,0.01]  *0.067* | 0.04[-0.04,0.12]  *0.323* | 0.00[-0.16,0.16]  *0.977* | 0.14[0.02,0.27]  ***0.026*** | |  |
| Fasting Insulin (mU/l) | -0.82[-1.68,0.04]  *0.061* | -0.02[-0.72,0.67]  *0.946* | 4.11[2.81,5.42]  ***<0.0001*** | 3.09[1.99,4.19]  ***<0.0001*** | |  |
| HOMA-IR | -0.17[-0.35,0.01]  0.061 | 0.02[-0.13,0.16]  *0.832* | 0.84[0.56,1.11]  ***<0.0001*** | 0.66[0.43,0.89]  ***<0.0001*** | |  |
| Body Fat Depots | | | | | |  |
| SAT Volume (cc) | -240.10[-384.93, -95.27]  ***0.001*** | 127.94[24.41,231.47]  ***0.015*** | 1619.60[1443.81,1795.40]  ***<0.0001*** | | 1113.80[950.45,1277.16]  ***<0.0001*** | |
| VAT Volume (cc) | -7.67[-36.51,21.17]  *0.602* | 6.24[-14.38, 26.85]  *0.553* | 235.83[200.82,270.83]  ***<0.0001*** | 168.50[135.97,201.03]  ***<0.0001*** | |  |
| Liver Fat (% weight) | 0.06[0-0.35,0.46]  *0.788* | 0.08[-0.21,0.36]  *0.600* | 1.32[0.82,1.82]  ***<0.0001*** | 0.86[0.41,1.30]  ***<0.0001*** | |  |
| IMCL (% water signal) | -0.11[0.38,0.16]  *0.432* | -0.01[-0.20,0.17]  *0.896* | 0.04[-0.28,0.36]  *0.813* | -0.05[-0.34,0.25]  *0.757* | |  |
| Covariates for adjusted models: ethnicity, ppBMI, sex, maternal education, rate of GWG (IOM), GDM, parity  *Values indicate odds ratio from binary logistic regression due to dichotomous outcome | | | | | |  |

Table S5 Abdominal Fat accumulation measures by MRI in early infancy (mean ± standard deviation corresponding to 300 children). SAT: Subcutaneous Adipose tissue, IAT: Intra-abdominal adipose tissue

|  | **Stable**  **Low** | **Stable**  **Normal** | **Stable**  **High** | **Early**  **Acceleration** | **Late Acceleration** |
| --- | --- | --- | --- | --- | --- |
| SAT (cc) | 84.60±24.78 | 90.93±25.06 | 94.25±23.45 | 112.93±34.19 (<*0.0001*)* | 87.04±25.72 |
| IAT(cc) | 20.89±7.05 | 22.55±6.71 | 23.94±8.89 | 28.1±11.90(*0.001*)* | 21.58±6.34 |
| *Significant Bonferroni corrected P value of comparison with Stable Normal group | | | | | |

Table S6. Unadjusted Behavioral and Cardio-metabolic assessments in childhood corresponding to different trajectory groups are presented in the table below (mean± standard deviation).

|  | **N** | **Stable**  **Low** | **Stable Normal** | **Stable**  **High** | **Early Acceleration** | **Late Acceleration** |
| --- | --- | --- | --- | --- | --- | --- |
| **Obesogenic eating behavior at 4.5 years** | | | | | | |
| Eating Rate (g/min) | 285* | 5.86±2.83 | 6.66± 3.42 | 7.18 ± 3.42 | 7.31 ± 3.22 | 8.51 ± 2.99 |
| Chews/g |  | 11.86± 6.69 | 10.96± 7.05 | 10.25 ± 7.39 | 10.97 ± 8.47 | 7.13 ± 2.90 |
| Oral Exposure per bite (s) |  | 19.74 ± 10.61 | 17.66 ± 11.38 | 19.04 ± 13.42 | 16.62 ± 6.56 | 19.24 ± 13.46 |
| Bite size (g/bite) |  | 1.75 ± 0.94 | 1.84 ± 1.22 | 2.07 ± 1.43 | 1.95 ± 0.89 | 2.48 ± 1.42 |
| Energy Intake, solids (kcal) |  | 207.55 ± 95.09 | 235.00± 127.88 | 250.84 ± 143.41 | 284.98 ± 151.20 | 290.70 ± 156.05 |
| Energy Intake, total (kcal) |  | 247.55 ±95.09 | 289.91± 145.70 | 311.06± 169.87 | 341.30 ± 141.01 | 350.80 ± 169.32 |
| Eating in Absence of Hunger (kcal) | 290** | 47.54 ±44.41 | 34.35 ± 42.55 | 42.95± 47.48 | 34.91 ± 33.50 | 45.58 ± 49.43 |
| **Cardio-metabolic outcome at 6 years** | | | | | | |
| Pre-hypertension |  |  |  |  |  |  |
| *No*  *Yes* | 644 | 75(91.5) | 244(86.5) | 152(81.7) | 25(69.4) | 41(70.7) |
|  |  | 7(8.5) | 38(13.5) | 34(18.22 | 11(30.6) | 17(29.3) |
| Fasting plasma glucose (mmol/l) | 485 | 4.44±0.36 | 4.52±0.38 | 4.58±0.39 | 4.56±0.25 | 4.68±0.31 |
| Fasting plasma insulin (mU/l) | 383 | 3.30±1.74 | 4.06±2.15 | 4.02±2.60 | 9.30±6.44 | 6.86±4.56 |
| HOMA-IR | 383 | 0.66±0.37 | 0.82±0.45 | 0.84±0.62 | 1.92±1.44 | 1.43±0.93 |
| **Fat accumulation measures from MRI scans at 6 years** | | | | | | |
| SAT(cc)  VAT(cc)  Liver Fat (% weight)  IMCL (% water) | 406 | 379.34±120.06 | 507.31±207.05 | 661.86±280.81 | 2241.18±1162.10 | 1661.85±611.97 |
|  |  | 166.81±46.00 | 170.85±45.27 | 180.79±44.41 | 411.50±193.40 | 325.85±138.69 |
|  | 378 | 0.55±0.74 | 0.51±0.49 | 0.59±0.57 | 1.90±2.98 | 1.22±1.23 |
|  | 375 | 0.56±0.82 | 0.55±0.73 | 0.55±0.60 | 0.64±0.37 | 0.57±0.38 |

*OE_min >2.99 **OE_min > 2.99 & Hunger_4.5_after > 1

Table S7. Mean±standard deviation of fetal abdominal circumference velocity (FACV) and birth weight and of LA and SNH using independent sample T-test

| Parameter | SNH | LA | p-value of significance of difference |
| --- | --- | --- | --- |
| FACV | 10.78±0.93 | 10.24±0.80 | <0.0001 |
| Birth Weight | 3.30±0.39 | 3.04±0.39 | <0.0001 |

Table S8. Binary logistic regression models were constructed to evaluate the odds of being classified in LA trajectory relative to SNH trajectory linked to early life predictors. Model-1 shows the association of maternal predictors (mutually adjusted). Model-2 shows association of offspring polygenic risk for obesity, adjusted for ethnicity and sex. Model-3 shows the association of breastfeeding duration, adjusted for ethnicity, ppBMI, education and GDM. Odds ratio [95%CI] with respect to the reference trajectory (stable normal) are presented. P values are indicated below each reported odds ratio. P values <0.05 are highlighted in bold., SNH: Stable Normal High, LA: Late Acceleration. Estimates from both complete case analysis and with multiple imputation are shown.

| Exposures | LA vs. SNH  (imputed) | | LA vs. SNH  (complete case) |
| --- | --- | --- | --- |
| Model-1: Influence of maternal antenatal factors (mutually adjusted) | | | |
| Race | |  |  |
| *Malay vs. Chinese*  *Indian vs. Chinese* | | 0.77[0.37,1.61]  *0.482* | 0.78[0.35,1.74]  *0.548* |
|  |  | 3.88[1.98,7.58]  ***<0.0001*** | 3.31[1.57,6.98]  ***0.002*** |
| Maternal Education | |  |  |
| *Secondary vs. University*  *Post-secondary vs. University* | | 1.43[0.69,2.98]  *0.334* | 1.38[0.63,3.02]  *0.420* |
|  |  | 1.12[0.57,2.20]  *0.748* | 1.04[0.50,2.16]  *0.919* |
| Parity | |  |  |
| *Nulliparous vs. Parous* | | 1.88[1.02,3.44]  ***0.042*** | 2.16[1.11,4.21]  ***0.023*** |
| Maternal Age (years) | | 1.01[0.95,1.07]  *0.801* | 1.01[0.95,1.08]  *0.725* |
| Pre-pregnancy BMI (kg/m^2^) | | 1.05[0.99,1.12]  *0.134* | 1.05[0.98,1.12]  *0.176* |
| Maternal height (cm) | | 1.01[0.96,1.06]  *0.821* | 1.01[0.96,1.08]  *0.798* |
| Gestational diabetes mellitus | |  |  |
| *Yes vs. No* | | 1.30[0.64,2.65]  *0.474* | 1.29[0.62,2.69]  *0.502* |
| Hypertensive disorders of pregnancy | |  |  |
| *Yes vs. No* | | 1.15[0.38,3.44]  *0.804* | 1.15[0.37,3.58]  *0.816* |
| Rate of GWG | |  |  |
| *Inadequate vs. Adequate*  *Excessive vs. Adequate* | | 1.43[0.56,3.67]  *0.455* | 1.70[0.66,4.39]  *0.275* |
|  |  | 1.01[0.50,2.04]  *0.975* | 1.17[0.57,2.40]  *0.672* |
| Model -2: Influence of offspring polygenic risk score for obesity (adjusted for ethnicity & sex) | | | |
| Polygenic Risk Scores (Obesity) | | 1.41[0.78,2.53]  *0.257* | 1.38[0.77,2.47]  *0.284* |
| Model- 3: Influence of breastfeeding duration (adjusted for ethnicity, ppBMI, education & gestational diabetes) | | | |
| Breastfeeding duration  *<3m Vs ≥3 months* | | 1.41[0.80,2.47]  *0.232* | 1.39[0.76,2.54]  *0.284* |

Table S9. Adjusted mean differences (β [95%CI]) in eating behaviors measured at age 4.5y between late acceleration and stable normal high trajectories (SNH class used as reference group), adjusted for ethnicity, ppBMI, sex, maternal education, rate of GWG (IOM), GDM, and parity. The stable normal trajectory pattern was used as the reference group for all models. Each row represents a separate model. P values are reported below each effect size measure. P values <0.05 are highlighted in bold. SNH: Stable Normal High, LA: Late Acceleration. Estimates from both complete case analysis and with multiple imputation are shown.

|  | LA Vs SNH  (imputed) | LA Vs SNH  (Complete case) |
| --- | --- | --- |
| Eating Rate (g/min) | 0.79[-0.91, 2.49]  *0.363* | 1.10[-0.59, 2.78]  *0.203* |
| Chews/g | -3.48[-6.96, -0.00]  ***0.05*** | -3.88[-7.56, -0.20]  ***0.039*** |
| Oral Exposure per bite (s) | 0.08[-7.00 7.16]  *0.983* | -0.67[-7.97,6.63]  *0.857* |
| Bite size (g/bite) | 0.33[-0.40 1.06]  *0.376* | 0.41[-0.23, 1.05]  *0.212* |
| Energy Intake, solids (kcal) | 37.49[-39.02,113.99]  *0.337* | 49.31[-25.51,124.14]  *0.196* |
| Energy Intake, total (kcal) | 35.61 [-52.54,123.76]  *0.429* | 51.88[-35.30, 138.05] *0.243* |
| Eating in Absence of Hunger (kcal)^a^ | -2.59[-28.73 23.56]  *0.846* | -0.17[-26.98, 26.65]  *0.990* |
| Covariates for adjusted models: sex, ethnicity, maternal education, ppBMI, rate of GWG (IOM), GDM, parity. ^a^Children with energy intake of less than 50 kcal in the eating in the absence of hunger assessment were excluded. | | |
